# Analysis of a Long Non-coding RNA associated Signature to Predict Survival in Patients with Bladder Cancer

**DOI:** 10.7759/cureus.24818

**Published:** 2022-05-08

**Authors:** Wenwen Zhong, Hu Qu, Bing Yao, Dejuan Wang, Jianguang Qiu

**Affiliations:** 1 Department of Urology, The Sixth Affiliated Hospital of Sun Yat-sen University, Guangzhou, CHN

**Keywords:** predict, the cancer genome atlas, bladder cancer, survival, long non-coding rna

## Abstract

Background: The study aimed to find a potential long non-coding RNA (lncRNA) model related to survival in bladder cancer by analyzing data in The Cancer Genome Atlas (TCGA).

Methods: We downloaded the gene expression data from the TCGA and analyzed the differentially expressed lncRNAs (DELs) between tumor and normal tissues. Patients were divided into training and testing groups, and a prognostic risk score model with lncRNAs was constructed by using data in the training group using multivariate Cox and lasso regression analysis. We divided patients into high-and low-risk groups according to the median value in the lncRNA signature model. Survival and receiver operating characteristic (ROC) curves were visualized in both groups. Further, we validated the model in the testing group.

Results: We screened 169 DELs for bladder cancer. The univariate Cox regression analysis showed that 13 lncRNAs were associated with prognosis with a *p-*value <0.01. We selected 12 of these lncRNAs to perform a multivariate Cox analysis to build the lncRNA signature. The formula with nine lncRNAs, namely, MIR497HG, LINC00968, NALCN-AS1, LINC02321, RNF144A-AS1, MNX1-AS1, FLJ22447, LINC01956, FLJ42969, was significantly related to prognosis. Patients in the high-risk group had a lower survival rate compared with the low‐risk group in the training and testing sets (both p*-*values < 0.05) and the area of the ROC curve was 0.737 and 0.68, respectively.

Conclusions: The study illustrated a significant lncRNA signature and indicated the risk score Cox model could be an important biomarker to predict the prognosis of bladder cancer.

## Introduction

Bladder cancer is one of the common types of malignant tumors, causing heavy social and economic burdens to the public. The incidence of bladder cancer ranks 10th among tumors and 6th among men, and it has the 9th highest mortality rate in the world [1]. With the aging of the population, changes in living habits and the environment and the popularization of cancer screening, the incidence and mortality rates of bladder cancer show a relatively stable status year by year in China [2]. Bladder cancer still exhibits the features of high aggressiveness and malignancy, and the majority of patients are usually diagnosed and treated at the advanced disease stage with unfavorable outcomes [3]. In the past decades, treatments for bladder cancer have rapidly improved. However, the overall survival (OS) time of bladder cancer patients has not been significantly extended, especially for patients with muscular invasive bladder cancers, with high malignancy and an extremely low 5-year survival rate of approximately 20-40% after radical surgery [4]. Recently, molecular biomarkers with prognostic and predictive values in bladder cancer have been illustrated due to the development of next-generation sequencing and gene expression profiling [5]. Additionally, long non-coding RNAs (lncRNAs) play a crucial role in predicting clinical outcomes [6]. However, a prognostic model based on the expression of lncRNAs has scarcely been studied. Therefore, it is critical to identify a novel lncRNA signature to better predict the prognosis of patients with bladder cancer and to provide potential targeted strategies.

LncRNAs, representing the largest and most diverse class of non-coding transcripts, are transcripts with lengths of more than 200 nucleotides with little or no protein-coding ability [7-8]. They were redundant segments of the genome but were considered useless transcripts in the past. However, in recent years, emerging studies have indicated the importance of lncRNAs in multiple cellular physiological and pathological processes [9-10]. Increasing evidence suggests that lncRNAs inhibit or activate gene expression by interacting with transcription factors or by binding to specific regions of mRNAs [11]. The roles and mechanisms of lncRNAs and their interactions with target genes have attracted the attention of scholars and represent a popular topic in cancer research. Researchers have identified lncRNA signatures in the prognosis of many cancers, including hepatocellular carcinoma and colorectal cancer. For bladder cancer, however, there are few published papers focusing on lncRNA signatures associated with prognosis.

In this study, we analyzed data from The Cancer Genome Atlas (TCGA) and aimed to find a potential long non-coding RNA (lncRNA) model related to survival in bladder cancer patients. RNA-seq data of bladder cancer were downloaded from The Cancer Genome Atlas (TCGA) official website to further analyze the characteristics of lncRNAs. We screened the differentially expressed lncRNAs (DELs) between tumor and normal tissues. Furthermore, we divided patients between the training and testing sets and performed Cox analysis to identify potential lncRNAs to construct the lncRNA risk score model to predict prognosis in bladder cancer. By dividing patients into high-and low-risk groups according to the median risk score, survival and receiver operating characteristic (ROC) curves as well as heatmaps were visualized. An identical analysis was repeated in the testing group to validate the Cox formula. In this work, we hope to determine the prognostic lncRNAs that may provide promising value to the prognosis and progression of bladder cancer in future research.

## Materials and methods

TCGA dataset

**Figure 1:**
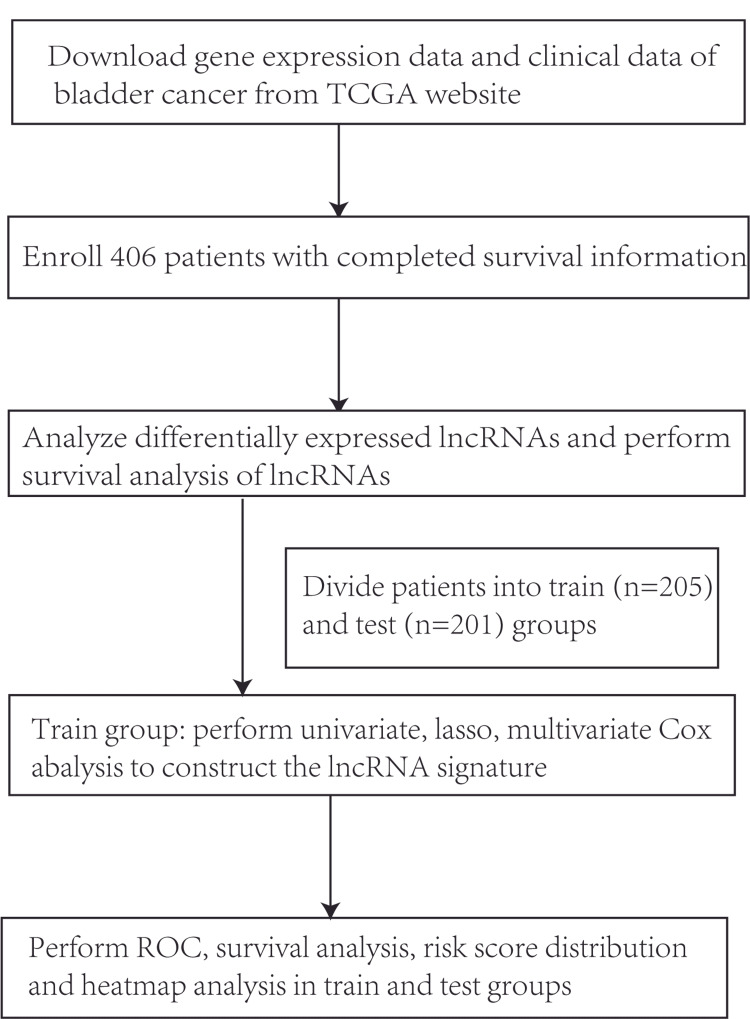
Flow chart of the study.

We downloaded a dataset containing the transcriptome profiles of RNA-seq data with gene expression quantification including 406 patients with information on survival time and vital status from the TCGA database on July 09, 2019. Furthermore, we screened the DELs between normal and tumor tissues in bladder cancer. In addition, we evaluated the correlation between lncRNA expression and OS rates in bladder cancer patients. Patients were divided into the training set (n=205) to construct the lncRNA signature and the testing set (n=201) to verify the lncRNA signature constructed with the multivariate Cox analysis. The flow chart of study is shown in Figure 1.

Identification of DELs

By using the “limma” package in R project (version 3.5.2; https://bioconductor.org/packages/limma), we performed differential expression analysis to identify DELs with the criteria of an adjusted p-value < 0.05 and a |log2 fold change (log2FC)| > 2 between tumor and adjacent normal tissues. Moreover, we used the “survival” package in R to further explore the association of lncRNAs with OS.

Selection of lncRNAs related to prognosis in bladder cancer

We divided patients into the training set (n=204) and the testing set (n=201) by using the R project. We performed a univariate Cox regression analysis to discover the DELs that were significantly related to OS with a p-value <0.01. Thirteen lncRNAs were selected as a result. Next, we applied lasso regression analysis to prevent overfitting of the model and to identify the most valuable lncRNAs to perform the multivariate Cox analysis. Finally, 12 lncRNAs were screened. A multivariate Cox analysis was conducted to construct the lncRNA signature by using the 12 lncRNAs found in the lasso regression model. Ultimately, a nine-lncRNA signature was identified.

Establishment of the risk score of the lncRNA signature

In the present study, the risk score model was constructed as follows: risk score = expression of DEL1 × β1 DEL1 + expression of DEL2 × β2 DEL2 +…expression of DELn × βn DELn. After the lncRNA prognostic model was found, we divided patients into high-and low-risk groups according to the median lncRNA expression-based risk score. The survival rate in both groups was compared with the Kaplan‐Meier survival analysis. Then, the receiver operating characteristic (ROC) curve was plotted. To reveal the sensitivity and specificity of the risk score model to predict the prognosis of patients with bladder cancer, the area under the curve (AUC) of the ROC curve was also calculated in the R project.

Validation of the lncRNA signature

To further determine the risk score model’s independent impact on the prognosis of bladder cancer, risk score distribution, patient vital status, and a heatmap of these nine lncRNA profiles were further examined using the R project. The testing set data was applied to verify the prognostic value of the nine-lncRNA signature model.

## Results

Screening of DELs

**Figure 2:**
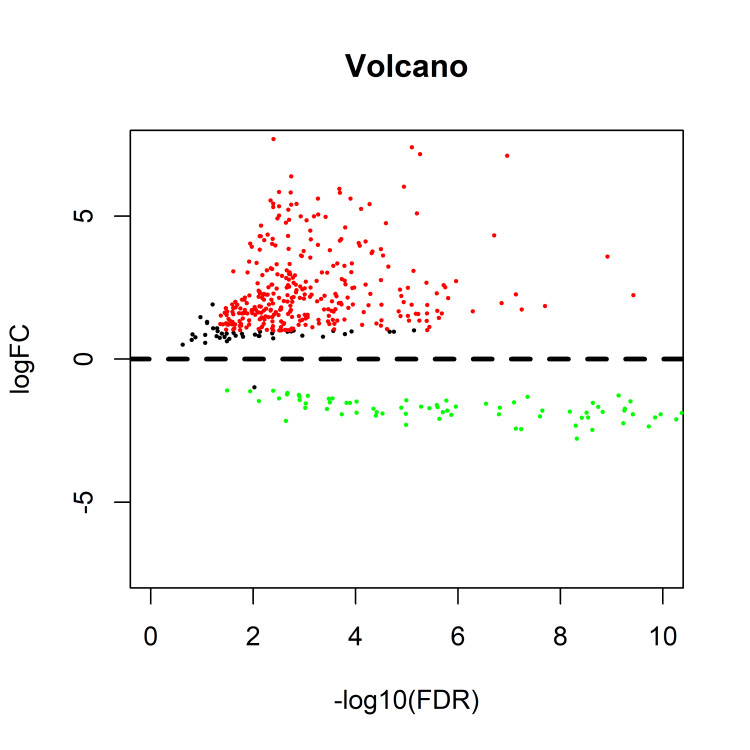
Volcano plot of the differentially expressed lncRNAs between tumor and normal tissues in bladder cancer The red dots indicate high-expression lncRNAs and the green ones indicate low- expression lncRNAs. The red ones demonstrate the lncRNA expression with both the log2FC > 2 and adjusted p-value < 0.05. FC: fold change; FDR: false discovery rate.

In this study, we included 406 samples with complete survival information. With the threshold definition of an adjusted p-value < 0.05 and a |log2 fold change (log2FC)| > 2, we eventually screened 169 DELs between the bladder cancer tumor and normal tissues. As a result, 116 lncRNAs were overexpressed in tumor tissues, while only approximately one-third of the lncRNAs were downregulated, as shown in the volcano plot (Figure 2).

**Figure 3:**
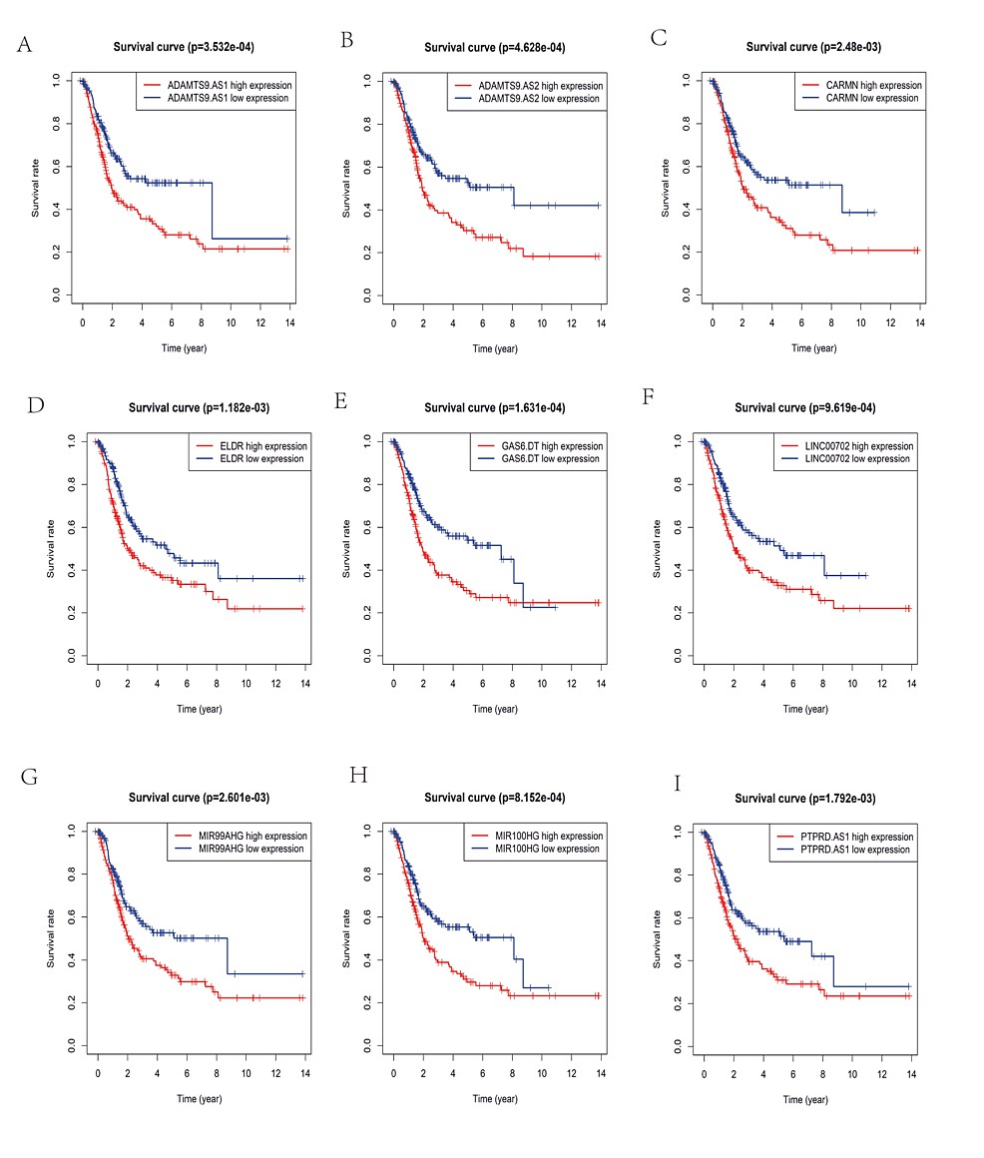
Survival curve of the top nine differentially expressed lncRNAs in bladder cancer (A) The relationship of ADAMTS9.AS1 expression with overall survival (B) The relationship of ADAMTS9.AS2 expression with overall survival (C) The relationship of CARMN expression overall survival (D) The relationship of ELDR expression with overall survival (E) The relationship of GAS6.DT expression with overall survival (F) The relationship of LINC00702 expression with overall survival (G) The relationship of MIR99AHG expression with overall survival (H) The relationship of MIR100HG expression with overall survival (I) The relationship of PTPRD.AS1 expression with overall survival.

And the top nine lncRNAs most relevant to survival prognosis are visualized in Figure 3.

Construction of a lncRNA-related prognostic signature

Thirteen lncRNAs in the univariate Cox regression analysis were significantly related to OS, with a p-value <0.01 in bladder cancer patients, as shown in Table 1.

**Table 1:** Univariate cox analysis of 13 lncRNAs with p-value <0.01

Gene	HR	HR.95L	HR.95H	p value
FLJ42969	1.005	1.002	1.007	9.15E-05
RNF144A-AS1	1.003	1.002	1.005	0.000
LINC00968	1.007	1.004	1.011	0.000
FLJ22447	1.000	1.000	1.001	0.000
LINC01956	1.011	1.004	1.018	0.001
LINC02321	1.022	1.008	1.035	0.001
GAS1RR	1.009	1.003	1.014	0.001
MIR100HG	1.000	1.000	1.001	0.003
NALCN-AS1	1.014	1.004	1.023	0.004
MNX1-AS1	1.002	1.001	1.004	0.004
LINC02525	1.004	1.001	1.007	0.005
MIR497HG	1.006	1.002	1.010	0.007
LINC02544	1.001	1.000	1.002	0.007

**Figure 4:**
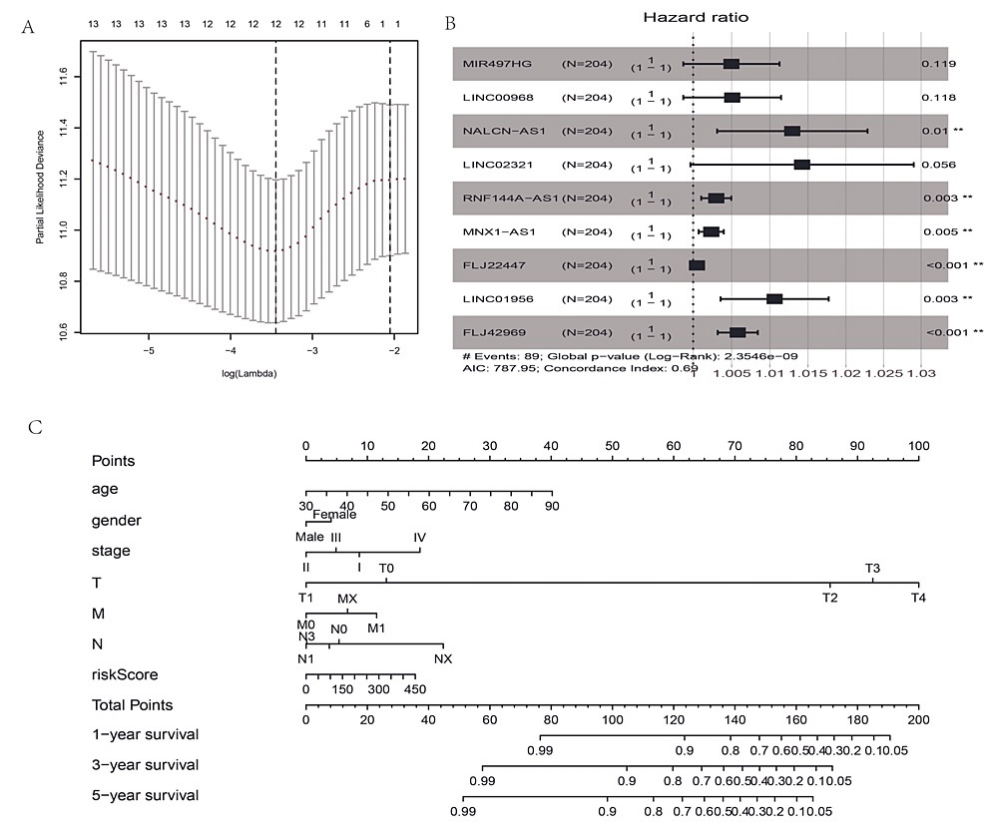
The Cox analysis results and survival nomogram (A) Lasso regression analysis of lncRNAs in univariate cox analysis. (B) The forest plot of the prognosis-related lncRNAs in the multivariate cox result. (C) Survival nomogram based on the risk score value of 369 patients with age, gender, stage, TNM stage parameters. TNM is a standard for classifying the extent of the spread of cancer. T describes the size of the original (primary) tumor and if it has invaded nearby tissue; N is to describe nearby (regional) lymph nodes that are involved; M describes distant metastasis (spread of cancer from one part of the body to another).

Lasso regression analysis was performed to identify the optimal lncRNAs to construct the lncRNA signature. Finally, 12 lncRNAs were screened in the lasso regression model (Figure 4A). Then, a multivariable Cox analysis was applied to identify the prognostic lncRNAs among the 12 candidate lncRNAs. Importantly, nine-lncRNA signatures, including MIR497HG, LINC00968, NALCN-AS1, LINC02321, RNF144A-AS1, MNX1-AS1, FLJ22447, LINC01956, and FLJ42969, was identified to be significantly associated with prognosis based on the Cox model, as shown in Figure 4B. The risk score model composed of the nine lncRNAs was constructed as follows: risk score = (0.0050 × expression level of MIR497HG) + (0.0051 × expression level of LINC00968) + (0.0129 × expression level of NALCN-AS1) + (0.0141 × expression level of LINC02321) + (0.0030 × expression level of RNF144A-AS1) + (0.0023 × expression level of MNX1-AS1) + (0.0005 × expression level of FLJ22447) + (0.0106 × expression level of LINC01956) + (0.0059 × expression level of FLJ42969). The nomogram (Figure 4C) was drawn to show the risk score value along with other clinical parameters to predict the survival rate in bladder cancer.

As the coefficient of the lncRNA derived from the multivariable Cox model is demonstrated in Table 2,

**Table 2:** The multivariate cox analysis of nine long non-coding RNAs associated with overall survival of bladder cancer patients

lncRNA	coef	HR	HR.95L	HR.95H	p-value
MIR497HG	0.0050	1.0050	0.9987	1.0113	0.1194
LINC00968	0.0051	1.0051	0.9987	1.0115	0.1178
NALCN-AS1	0.0129	1.0129	1.0031	1.0229	0.0098
LINC02321	0.0141	1.0142	0.9996	1.0290	0.0558
RNF144A-AS1	0.0030	1.0030	1.0010	1.0050	0.0026
MNX1-AS1	0.0023	1.0023	1.0007	1.0040	0.0046
FLJ22447	0.0005	1.0005	1.0003	1.0007	1.48E-05
LINC01956	0.0106	1.0106	1.0036	1.0177	0.0032
FLJ42969	0.0059	1.0058	1.0032	1.0084	1.29E-05

**Figure 5:**
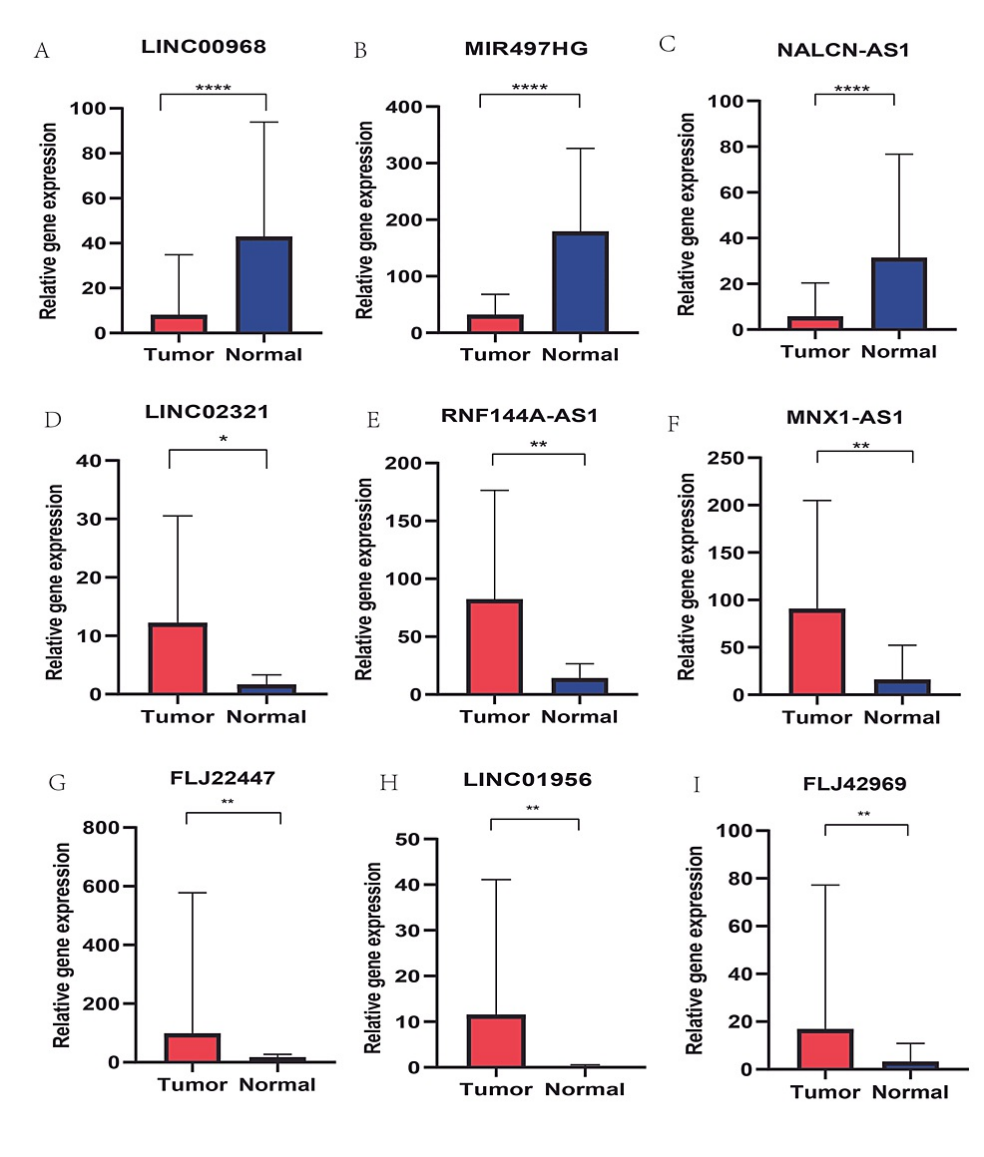
The expression level between tumor and normal tissues of the nine lncRNAs in the lncRNA signature of bladder cancer (A) The expression level of LINC00968 between tumor and normal tissues in bladder cancer (B) The expression level of MIR497HG between tumor and normal tissues in bladder cancer (C) The expression level of NALCN-AS1 between tumor and normal tissues in bladder cancer (D) The expression level of LINC02321 between tumor and normal tissues in bladder cancer (E) The expression level of RNF144A-AS1 between tumor and normal tissues in bladder cancer (F) The expression level of MNX1-AS1 between tumor and normal tissues in bladder cancer (G) The expression level of FLJ22447 between tumor and normal tissues in bladder cancer (H) The expression level of LINC01956 between tumor and normal tissues in bladder cancer (I) The expression level of FLJ42969 between tumor and normal tissues in bladder cancer

Furthermore, the relative expression levels of these nine lncRNA between tumor and normal tissues were compared, as shown in Figure 5.

**Figure 6:**
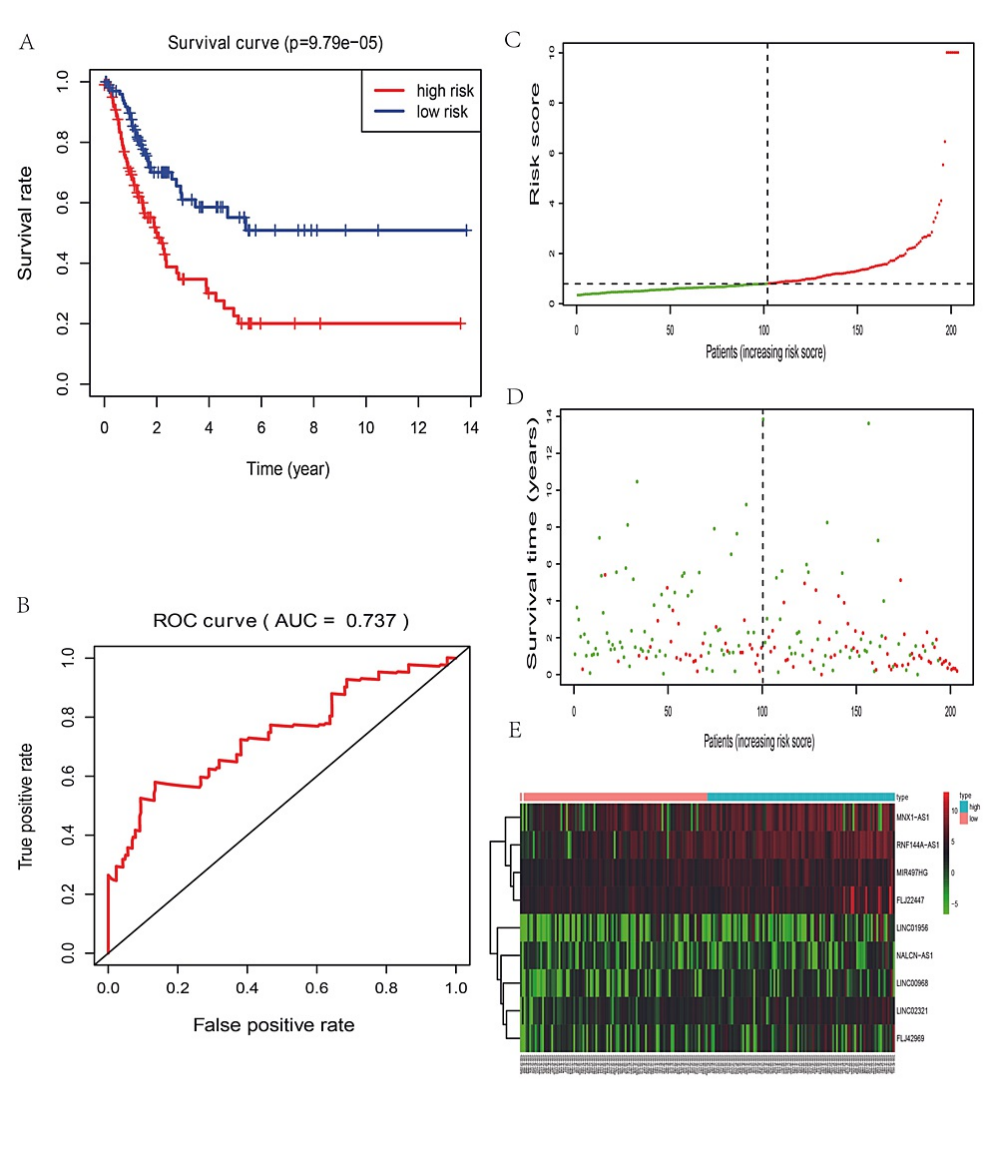
The overall survival prediction of the nine long non-coding RNA signature-based risk score in bladder cancer in the training set (n = 204) (A): Kaplan-Meier analysis of patients’ overall survival in the high-risk and low-risk subgroups; (B): The receiver operating characteristic (ROC) analysis of the risk score to predict the overall survival. (C): The risk score distribution based on the nine-lncRNA signature; (D): patient survival status and duration based on the nine-lncRNA signature risk score; (E): Heatmap of the nine-lncRNA expression profiles in the high risk and low-risk subgroups in the training set. The red color in figure 6D means dead status, the green one means alive status. The color trend from red to blue in figure 6E demonstrates a trend from low expression to high expression.

As a result, the nine lncRNAs were differentially expressed, with a p-value < 0.05. By using the score model, we calculated the risk score for each patient in the training group and divided the training group into a high-risk group (n = 102) and a low-risk group (n = 102) according to the median risk score. In the training set, the high-risk group had a lower survival rate compared with the low‐risk group, with approximate 3-year and 5-year survival rates of 34.7% and 22.6%, respectively, in the high-risk group, which were lower than those in the low-risk group (61.0% and 50.9%, respectively) (p-value = 9.79e-05, Figure 6A). In addition, the area of the ROC curve for the nine-lncRNA signature in the training set was 0.737, which indicated a good performance (Figure 6B). The patients’ risk scores ranged considerably (Figure 6C), and the survival status is described on the dot plot (Figure 6D). From the information in the plots, we can tell that patients with a high-risk score had a low survival rate, implying that the mortality rate in the high-risk group was higher than that in the low-risk group. The heatmap of these nine lncRNAs shown in Figure 6E revealed that MNX1-AS1, RNF144A-AS1, MIR497HG, and FLJ22447 were upregulated in the high-risk group with positive coefficients, while the other lncRNAs seemed to have similar but less obvious trends.

Verification of the lncRNA signature in the testing set

**Figure 7:**
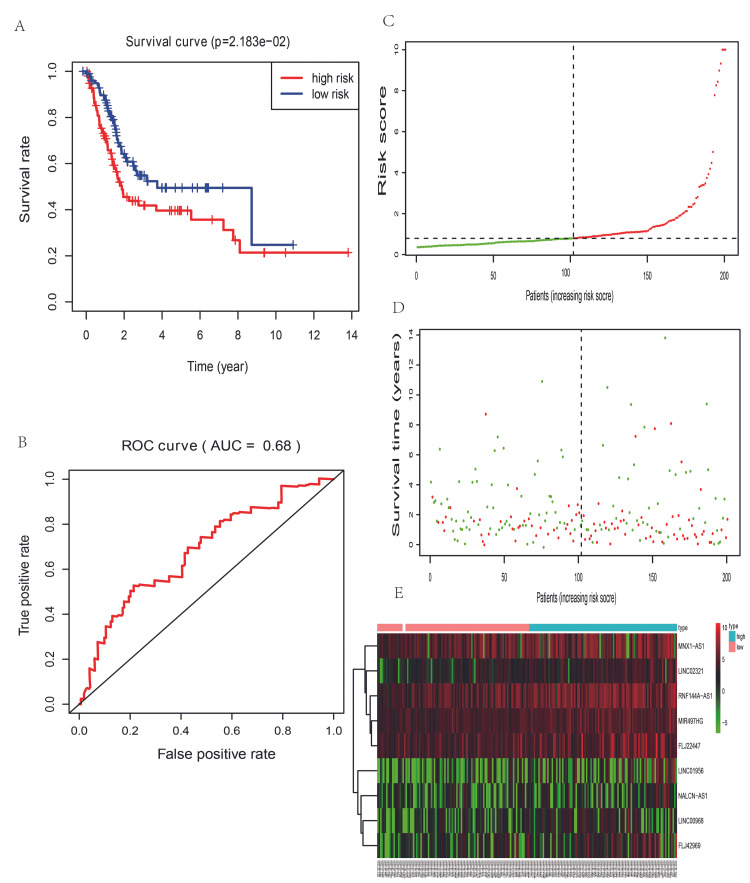
The overall survival prediction of the nine-long non-coding RNA signature-based risk score in bladder cancer in the testing set (n = 201) (A): Kaplan-Meier analysis of patients’ overall survival in the high-risk and low-risk subgroups; (B): The receiver operating characteristic (ROC) analysis of the risk score to predict the overall survival. (C): The risk score distribution based on the nine-lncRNA signature; (D): patient survival status and duration based on the nine-lncRNA signature risk score; (E): Heatmap of the nine-lncRNA expression profiles in the high risk and low-risk subgroups in the training set. The red color in figure 7D means dead status, the green one means alive status. The color trend from red to blue in figure 7E demonstrates a trend from low expression to high expression.

To verify the prognostic value of the nine-lncRNA signature, data in the testing set were used. First, we calculated the risk scores of patients in the test group according to the formula based on the expression of the nine lncRNAs and divided the test group into high-risk and low-risk groups using the same cutoff value as in the training group. As the results demonstrated, the survival rate in the low-risk group was much higher than that in the high-risk group (Figure 7A, p = 2.183e-02), similar to the result in the training set. The 3-year and 5-year survival rates were approximately 41.8% and 35.7%, respectively, in the high-risk group, which were lower than those in the low-risk group. The AUC for the nine-lncRNA-based risk score in the testing group was 0.68 (Figure 7B). Similar trend results were obtained in the risk score distribution (Figure 7C), patient vital status (Figure 7D), and heatmap of the nine lncRNA profiles (Figure 7E). The results in the testing set illustrated that the nine-lncRNA signature could reasonably reflect the prognosis of bladder cancer.

## Discussion

Bladder cancer is one of the most common and significant cancers with multiple potential risk factors, including smoking and chemical exposure [12]. Accumulating investigations have been carried out to explore the pathogenesis and aggravation of bladder cancer [13-15]. An increasing number of studies have revealed that long non-coding RNAs play a crucial role in the carcinogenesis and progression of bladder cancer through various mechanisms [16-20]. However, the specific regulation of lncRNAs and a related prognosis model in bladder cancer are still far from clear. The identification of a lncRNA-associated signature will help to predict the prognosis of bladder cancer and facilitate the development of novel therapeutic targets in further research.

In this study, we measured data in the TCGA to identify DELs and constructed a lncRNA-based signature related to prognosis in bladder cancer. Through an integrative analysis with bioinformatics methods, we identified a nine-lncRNA signature (including MIR497HG, LINC00968, NALCN-AS1, LINC02321, RNF144A-AS1, MNX1-AS1, FLJ22447, LINC01956, and FLJ42969) to predict the OS and prognosis of bladder cancer patients. In contrast to other diseases, there is limited research about the roles of these nine lncRNAs in bladder cancer. Several studies have found that the long noncoding RNA LINC00968 promoted cell proliferation and migration in different cancers through different mechanisms [21-24] and accelerated the progression of coronary artery disease [25] and diabetic nephropathy [26]. These findings suggest that LINC00968 is probably a positive risk factor in several diseases, which contradicts our result showing that LINC00968 was downregulated in tumor tissues. MIR497HG was identified as a hub lncRNA in ischemic stroke [27]. The lncRNA RNF144A-AS1 was reported as a prognostic factor of chemoresistance in ovarian cancer, while its expression level was downregulated, contradicting its expression in bladder cancer [28]. Additionally, RNF144A-AS1 was illustrated as a positive gene related to Alzheimer’s disease [29]. Ding et al. reported that FLJ22447 was remarkably upregulated in stromal carcinoma-related fibroblasts [30]. However, more studies need to be performed to explain the specific roles of these nine lncRNAs.

As the lncRNA signature was verified in the TCGA dataset, it will probably provide efficient values in practice. Nevertheless, there are limitations to the present work. First, it was a bioinformatic study with a limited sample size that may influence the lncRNA signature results. Second, experimental confirmation, including cell lines and animal studies, has been lacking thus far. There are limited publications exploring the unique mechanism of each lncRNA of the nine lncRNAs. We propose further research on the potential mechanism of each lncRNA in the model and the use of clinical samples to verify the accuracy of the lncRNA signature.

## Conclusions

We analyzed the TCGA dataset with bioinformatics methods including differential analysis, lasso and Cox regression analysis. The present study illustrated a valuable lncRNA signature to predict the prognosis of bladder cancer and the risk score based on the signature could be an important biomarker for future studies. Meanwhile, further research on the potential mechanism of each lncRNA in the model is necessary and clinical samples would be used to verify the accuracy of lncRNA signatures next.
